# Impact of acute exercise on peripheral blood mononuclear cells nutrient sensing and mitochondrial oxidative capacity in healthy young adults

**DOI:** 10.14814/phy2.15147

**Published:** 2021-12-10

**Authors:** Bailey Theall, James Stampley, Eunhan Cho, Joshua Granger, Neil M. Johannsen, Brian A. Irving, Guillaume Spielmann

**Affiliations:** ^1^ School of Kinesiology Louisiana State University Huey P Long Fieldhouse Baton Rouge Louisina USA; ^2^ Pennington Biomedical Research Center Baton Rouge Louisina USA

**Keywords:** acute exercise, lymphocyte activation, oxidative phosphorylation, PBMC, respiration

## Abstract

Regular exercise is associated with changes in peripheral blood mononuclear cell (PBMC) proportions that have enhanced effector functions in young and old adults; however, the effects of acute exercise on PBMC nutrient sensors and metabolic function in active young adults is unknown. To fill this gap, activation status and nutrient‐sensing mechanisms of PBMCs isolated from 21 healthy active adults (20–35 yr; 36.5 ± 6.3 V̇O_2peak_) were characterized before and after 30 min of moderate‐to‐vigorous cycling (65%–75% V̇O_2peak_). In addition, changes in PBMC mitochondrial respiratory function in response to exercise were assessed using high‐resolution respirometry. There was an increase in the number of activated CD69+/CD4 (79% increase) and CD69+/CD8 (166% increase) T‐cells in response to the acute bout of exercise, while the nutrient‐sensing mechanisms remained unchanged. PBMC mitochondrial respiration did not increase on a cell‐per‐cell basis, however, mitochondrial oxidative capacity (OXPHOS) increased at the tissue level (18.6 pmol/(s*ml blood) versus 29.3 pmol/(s*ml blood); *p* < 0.05) in response to acute exercise. Thus, this study shows that acute exercise preferentially mobilizes activated T‐cells while concomitantly increasing PBMC mitochondrial oxidative capacity at the tissue level, rather than acutely changing mitochondrial oxidative capacity at the cellular level in young adults.

## Graphical abstract

Activation status and nutrient‐sensing mechanisms of PBMCs isolated from 21 healthy adults were characterized before and after 30 min of moderate‐to‐vigorous cycling. PBMC mitochondrial respiration did not increase on a cell‐per‐cell basis, however, mitochondrial oxidative capacity (OXPHOS) increased at the tissue level (18.6 pmol/(s*ml blood) vs. 29.3 pmol/(s*ml blood); *p* < 0.05) in response to acute exercise. Thus, this study shows that acute exercise preferentially mobilizes activated T‐cells while concomitantly increasing PBMC mitochondrial oxidative capacity at the tissue level, rather than acutely changing mitochondrial oxidative capacity at the cellular level in young adults.

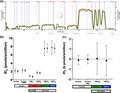

## 1 INTRODUCTION

Exercise has long been considered crucial for improving cardiometabolic health. However, recent research also highlights the benefits of exercise on improving immune function in adults of all ages (Duggal et al., 2019). Given the immune system's complexity, the mechanisms linking regular exercise to enhanced immune function are likely multi‐dimensional. Cross‐sectional studies have shown that individuals who participate in lifelong physical activity have healthier immune function than their sedentary counterparts, including lower pro‐inflammatory cytokine secretions (Duggal et al., 2018). In addition, lifelong exercisers also have lower concentrations of senescent T‐cells and concomitantly higher concentrations of naïve T‐cells (Spielmann et al., 2011). Emerging evidence suggests decreased metabolic function occurs with CD8+ T‐cell differentiation due to preferential use of glycolytic metabolism, potentially contributing to the pro‐inflammatory state commonly seen in older adults (Callender et al., 2020). Exercise is known to improve the metabolic function of muscle tissue by acutely increasing energy demand and requiring higher rates of ATP resynthesis (Egan & Zierath, 2013). While maintaining metabolic fitness appears to facilitate the proper functioning of T‐cells (Almeida et al., 2016), the effects of exercise on lymphocyte metabolic function have only recently been explored with mixed results (Palmowski et al., 2021; Siedlik et al., 2017).

Acute exercise stimulates lymphocyte activation (Curran et al., 2019), leading to rapid transitions from quiescence to cell growth, proliferation, and differentiation into functional effector cells (Pearce & Pearce, 2013). These transitions have considerable energy and biosynthetic demands, thus activated T‐cells undergo metabolic reprogramming to support these transitions (Geltink et al., 2018). Activated T‐cells can also upregulate the expression of nutrient transporters (Macintyre et al., 2014; Sinclair et al., 2013) to facilitate the uptake of energy‐rich nutrients while simultaneously increasing the concentration of downstream enzymes involved in ATP production (Tan et al., 2017). However, the impact of acute exercise on the expression of T‐cell nutrient sensors and bioenergetics is less clear. For example, a recent human study (Palmowski et al., 2021) performed in a small cohort of young males showed that the CD4+ T‐cell phenotype, activation, and oxygen consumption rates did not change after culturing the cells in the postexercise serum. Conversely, data suggest that an hour‐long bout of low exercise increases PBMC mitochondrial respiration, particularly for fatty acid oxidation measured using high‐resolution respirometry (Liepinsh et al., 2020). Moreover, some (Busquets‐Cortes et al., 2017; Rosa et al., 2021) but not all (Hedges et al., 2019) studies have shown PBMC mitochondrial content and/or function improvements with training. Thus, whether any phenotypic or metabolic changes in T‐cell subsets would occur in response to exercise remains unclear (Padilha et al., 2021).

Considering the limited and conflicting data, the purpose of this study was to characterize the effects of an acute, moderate‐to‐vigorous intensity bout of exercise on changes in PBMC composition, including cellular composition, expression of nutrient sensors, and mitochondrial respiration in healthy young adults. We hypothesized that (i) acute exercise would preferentially mobilize highly differentiated T‐cells with impaired OXPHOS (ii) the number of activated T‐cells would be greater postexercise compared to baseline, (iii) the number of T‐cells expressing nutrient sensors would also be greater postexercise compared to baseline, (iv) the number of activated T‐cells will be associated with the number of T‐cells expressing nutrient sensors, and (v) PMBC mitochondrial oxidative capacity would be lower postexercise compared to baseline, highlighting a greater reliance on glycolysis over OXPHOS.

## 2 MATERIALS AND METHODS

### 2.1 Participants

Twenty‐one healthy adults (age: 27.0 ± 5.4 years; women: 43%) were enrolled in this study (Table 1). Participants were excluded if they presented any metabolic or cardiovascular disease. One additional participant was enrolled in the study but was excluded from the present analysis due to technical difficulties. The study was conducted at the Louisiana State University (LSU), Baton Rouge, LA, USA. The study protocol was approved by the Institutional Review Board at the LSU, and all participants voluntarily provided signed, informed consent prior to their participation.

**TABLE 1:** Physical characteristics

	All (*n* = 21)	Females (*n* = 9)	Males (*n* = 12)
Age (years)	27.0 ± 5.4	24.0 ± 3.8	29.3 ± 5.4*
Height (cm)	171.8 ± 8.3	165.0 ± 6.7	176.9 ± 5.1*
Mass (kg)	76.5 ± 15.5	66.3 ± 6.4	84.1 ± 16.1*
BMI (kg/m^2^)	25.8 ± 4.0	24.4 ± 2.9	26.8 ± 4.5

Note

Mean ± SD; Sex differences indicated by * *p* < 0.05 using independent test.

Abbreviation: BMI, body mass index.

### 2.2 VO_2peak_ and experimental exercise protocol

All participants completed a graded exercise test and an acute, steady‐state exercise session on a Velotron cycle ergometer (Racermate, Inc.) separated by at least 2 days (maximum one week). On visit 1, a graded exercise test was used to determine peak oxygen uptake (V̇O_2peak_). Participants completed a standardized 5‐min warm‐up at 25 W and were given a 3‐minute break before beginning the exercise test. The workload increased by 25 W every 3 min until RER was >1.00; then, the workload increased by 25 W every 2 min. Participants were allowed to stop the exercise if one or more of the following situations was met: respiratory exchange ratio (RER) was ≥1.1 or higher; rating of perceived exertion (RPE) was ≥17 on the Borg Scale; heart rate (HR) was ≥90% of age‐predicted maximum (220 ‐ age); or the V̇O_2_ reached a plateau (i.e., V̇O_2_ did not increase with the increased workload). Respiratory gases were measured using an automated metabolic system (TrueOne 2400, ParvoMedics, Inc.), and respiratory gas measurements were averaged for 30‐s periods. HR was recorded every 5 minutes (Polar T31 coded transmitter, Polar Electro Oy, Finland).

On visit 2, the acute, steady‐state exercise bout was conducted. The participants reported to the laboratory following an overnight fast (>10 h). The participants completed a 30‐min steady‐state cycling protocol at a power output corresponding to 65%–75% of their V̇O_2peak_. HR and V̇O_2_ were monitored continuously, and the workload was adjusted to keep participants within the specified range. Intravenous blood samples were collected in 10 ml EDTA vacutainers (Becton, Dickinson, and Co.) before (after 5‐min of seated rest) and immediately after exercise. An additional blood sample was taken at rest in serum separating tubes (Becton, Dickinson, and Co.) to determine cytomegalovirus (CMV) serostatus. All blood samples were processed immediately for cell counting, peripheral blood mononuclear cell (PBMC) separation, and serum was stored at −80°C to determine CMV serostatus.

### 2.3 PBMC surface antigen labeling

Cell counts were determined using a hematology analyzer (Sysmex XN‐330, Sysmex Co.). PBMCs were isolated using density‐gradient centrifugation (Ficoll‐Paque; GE Healthcare Bio‐Sciences AB) as previously described (Theall et al., 2020). Isolated PBMCs (1 × 10^6^) were labeled with 100 *μ*l of prediluted fluorochrome‐labeled antibodies (Abs). Combinations of Abs were used to characterize the T‐cell level of differentiation and nutrient‐sensing mechanism, namely anti‐CD3‐FITC (clone OKT3), anti‐CD69‐PE (clone FN50), anti‐CD25‐PE (clone BC96), anti‐CD71‐PE (clone OKT9), anti‐CD38‐PE (clone HB7), anti‐CD4‐PE (clone OKT4), anti‐CD57‐PE (clone TB01), anti‐CD4‐PerCP‐cy5.5 (clone RPA‐T4), anti‐CD8‐PerCP‐cy5.5 (clone RPA‐T8), anti‐CD36‐PerCP‐cy7 (clone eBioNL07), anti‐CD3‐APC (clone OKT3), and anti‐CD8‐APC (clone OKT8, Thermo Fisher Scientific); anti‐killer cell lectin‐like receptor G1 (KLRG1)‐FITC (clone REA261, Miltenyi Biotech); anti‐GLUT‐1‐PE (clone 202915, R&D Systems); anti‐GLUT‐4‐PE (Biorbyt). All mAbs were previously titrated to determine optimal dilutions for flow cytometry. Cells were incubated for 45‐min in the dark at room temperature.

### 2.4 Intracellular staining

Isolated PMBCs were washed once with PBS then with PBS/1% BSA. Following washing, aliquots of 1 × 10^6^ PBMC were labeled with anti‐CD3‐FITC (clone OKT3), anti‐CD4‐PE (clone OKT4), and anti‐CD8‐PerCP‐Cy5.5 (clone RPA‐T8, Thermo Fisher Scientific). Following two washing steps, cells were fixed in 4% paraformaldehyde, permeabilized in saponin buffer, and incubated with anti‐HK‐1‐APC or anti‐HK‐2‐APC (AssayPro).

### 2.5 Flow cytometry

PBMC phenotype and intracellular expression were characterized by four‐color flow cytometry using an Accuri C6 flow cytometer (Accuri) as previously described (Theall et al., 2020). Flow cytometry analyses were conducted using BD Accuri C6 software (Version 1.0.264.21). Forward and side scatter characteristics were used to gate on the lymphocyte population and side scatter against CD3+ cells. CD4+ and/or CD8+ cells were gated within CD3+ cells, and expression of activation, nutrient, differentiation markers was assessed on CD3+CD4+ and CD3+CD8+ T‐cells. For each sample, 100,000 events in the lymphocyte gate were collected for analysis. The percentages of all CD3+CD4+ and CD3+CD8+ T‐cells expressing the markers of interest were tabulated for statistical analysis. Total cell numbers of each lymphocyte subset were determined by multiplying the percentage of all lymphocytes expressing the markers of interest by the total lymphocyte count.

### 2.6 Determination of CMV serostatus

Serum samples were obtained by centrifugation and frozen at −80°C until analysis for anti‐CMV IgG antibodies. IgG‐class antibodies against CMV were determined in duplicate by a quantitative enzyme immunoassay (SERION ELISA classic Cytomegalovirus IgG/IgM; Institut Virion/Serion, Würzburg, Germany) according to the manufacturer's instruction. Results were determined using a 96‐well microplate reader (Molecular Devices).

### 2.7 High‐resolution respirometry (HRR)

The PBMC respiratory rates were measured using HRR (Oroboros Oxygraph‐O2k; Oroboros Instruments). A standardized substrate inhibitor titration (SUIT) protocol was used to determine routine respiration rates in intact PBMCs, and respiration rates during LEAK (State 4), OXPHOS (State 3), and electron transfer system (ETS) capacity states in digitonin permeabilized PBMCs at 37°C and O_2_ concentrations between ~200 µM and 50 µM. After isolation, the PBMCs were resuspended in a mitochondrial respiration media (MiR05 Kit, Oroboros, Austria, 0.5 mM EGTA, 3 mM MgCl_2_, 60 mM Lactobionic Acid, 20 mM Tuarine, 10 mM KH_2_PO_4_, 20 mM HEPES, 110 mM Sucrose, 1 g/L Fatty Acid Free BSA, pH 7.1) and transferred into two independent oxygraph chambers for duplicate measurements (~4–8 million PBMCs per chamber). The starting reaction mixture consisted of MiR05 plus Amplex Ultrared (10 µM), horse‐radish peroxidase (1 U/ml), superoxide dismutase (SOD, 5 U/ml). After adding PBMC's to the chambers, H_2_O_2_ titrations (0.1 µM) were performed to calibrate the AmplexUltraRed H_2_O_2_ assay. After stabilization, routine respiration was measured in the absence and presence of pyruvate (5 mM), malate (2 mM), and glutamate (10 mM). Next, digitonin was added (approximately 7.5–10 µg/10^6^ cells) for 5–10 min to chemically permeabilize the plasma membranes of the PBMCs. In the presence of 5 mM pyruvate, 2 mM malate, and 10 mM glutamate and in the absence of ADP, NADH‐Linked (N‐Linked) LEAK respiration (LEAK_N_) was measured. NADH plus Succinate‐Linked (NS‐Linked) LEAK respiration (LEAK_NS_) was measured following the addition of 10 mM succinate. A saturating concentration of ADP‐Mg^++^ (5 mM) was added to stimulate NS‐Linked (N‐Linked) maximal oxidative phosphorylation (OXPHOS_NS_) capacity. Carbonyl cyanide m‐chlorophenyl hydrazine (CCCP), a chemical uncoupler, was titrated at ~1 µM per step to the maximal uncoupled respiration rates associated with ETS capacity. Finally, we added 2.5 µM antimycin A to measure residual oxygen consumption. The HRR data were acquired and analyzed using DatLab software (version 7.1, Oroboros Instruments). The residual oxygen consumption rates were subtracted from the routine LEAK, OXPHOS, and ET capacity rates to isolate the mitochondrial oxygen consumption rates during these respiratory states. The respiration rates were normalized per million cells (pmol/(s*10^6^ cells)), referred to as oxygen flow (*I*O_2_). Since acute exercise induces changes in circulating immune cell numbers and proportions, contrary to what is observed in muscle tissue, the respiration rates were normalized per ml of blood (pmol/(s*ml blood)) by multiplying the *IO_2_* by the concentration of PBMCs in 1 ml of blood, referred to as tissue oxygen flow (*I*O_2‐tissue_). The PBMCs are defined as leukocytes plus monocytes, were counted using a hematology analyzer and confirmed with flow cytometry. The respiratory rates were also corrected for daily room air calibrations as well as regular instrumental background and zero calibrations. Note: due to a technical issue with the SOD in the AmplexUltraRed H_2_O_2_ assay, the AmplexUltraRed H_2_O_2_ was not analyzed. As noted previously, one participant was excluded from the present analysis due to the low viability of the participant's cells during the HRR experiment, as indicated by the respiratory control ratio (OXPHOS_NS_/LEAK_NS_) <1.0 for the pre‐exercise sample. One sample out of 42 samples was run in singlet due to an insufficient number of cells for a duplicate measurement. The median coefficient of variations for the routine respiration, LEAK_NS,_ OXPHOS_NS,_ and ET capacity rates were ~11%, 12%, 7%, and 6%, respectively.

### 2.8 Statistical analysis

SPSS version 27 (Chicago, IL, USA) was used for all statistical analyses. Descriptive analysis such as frequency, mean, standard deviations, and medians were calculated prior to data analysis. T‐cell percentages and numbers were skewed at *p* < 0.05 significance level when checked using the Kolmogorov–Smirnov test. Therefore, nonparametric tests were used to analyze the change in participants’ T‐cell subsets. Wilcoxon signed‐rank tests were conducted to find the differences between resting and postexercise T‐cell percentages and numbers, and Mann–Whitney *U* tests were used to analyze the differences between sex and CMV serostatus at rest and post‐exercise. After assessing the normality of the T‐cell mitochondrial respiration data, statistically, significant differences in mitochondrial respiration rates after exercise were identified by using paired *t*‐tests. Independent *t*‐tests were used to compare sex differences for baseline characteristics, exercise performance measures, and T‐cell mitochondrial respiration rates in PBMCs at resting and post‐exercise. Spearman's correlation analysis was used to identify the correlation between various T‐cell subsets and mitochondrial respiration rates. Data are presented as mean ± standard deviation (SD) unless otherwise stated. Statistical significance was declared at *p* < 0.05 for all analyses.

## 3 RESULTS

### 3.1 Physiological response to exercise

The physical characteristics and exercise performance measures collected during the graded (maximal) exercise test and the steady‐state exercise bout are presented in Table 2. All participants completed the steady‐state exercise bout. Males achieved higher maximum power during the *VO_2peak_* exercise test and lower heart rates during the submaximal exercise test.

**TABLE 2:** Exercise performance measures

	All (*n* = 21)	Female (*n* = 9)	Male (*n* = 12)
Maximum exercise test			
Maximum power (W)	213.2 ± 36.3	189.3 ± 33.6	227.1 ± 31.1*
Maximum heart rate (HRmax)	185.8 ± 13.6	188.5 ± 13.1	183.9 ± 14.2
V̇O_2_peak (ml·kg^−1^*·min^−1^)	36.5 ± 6.3	36.8 ± 3.1	36.3 ± 8.1
Submaximal exercise test			
Mean V̇O_2_	25.0 ± 4.2	25.5 ± 3.3	24.7 ± 5.5
Mean V̇O_2_ (%V̇O_2_peak)	69.4 ± 14.4	68.8 ± 8.7	69.7 ± 17.6
Mean heart rate (HR)	156.6 ± 16.7	166.3 ± 15.2	150.1 ± 14.8*
Mean heart rate (%HRmax)	84.3 ± 6.9	88.4 ± 7.3	81.6 ± 5.4*

Note

Mean ±SD; Sex differences indicated by * *p* < 0.05 using independent *t*‐tests; V̇O_2_: rate of oxygen uptake.

### 3.2 Effect of exercise on T‐cell phenotype and nutrient sensing

Table 3 presents the CD4+ and CD8+ phenotype and expression of nutrient sensors at rest and post‐exercise across all participants. There was an increase in the percentage and number of naïve (KLRG1‐/CD57‐) and senescent (KLRG1+/CD57+) CD8+ T‐cells after the exercise bout; however, there was only an increase in the number of naïve CD4+ T‐cells (*p* < 0.05) after the exercise bout. The number of CD4+ T‐cells expressing glucose transporter 4, GLUT4, increased by 53% after exercise (*p* = 0.046). Additionally, the percentage of CD8+ T‐cells expressing fatty acid translocase, CD36 (pre: 10.05 ± 9.52% vs. post: 7.22 ± 5.95%. *p* = 0.007) and CD4+ T‐cells expressing hexokinase 1, HK1 (pre: 6.89 ± 22.03% vs. post: 2.55 ± 7.95%, *p* = 0.013), and hexokinase 2, HK2 (pre: 6.75 ± 21.43% vs. post: 6.20 ± 20.55%, *p* = 0.023), decreased after exercise. A significant 55% decrease in the number of CD4+HK1+ T‐cells was also observed after the exercise bout (*p* = 0.028). Similarly, the percentage CD8+ T‐cells expressing HK1 also decreased after exercise (pre: 13.52 ± 16.84% vs. post: 10.48 ± 15.06%, *p* = 0.033). Although the participant's sex had no effect on nutrient sensors, CMV seropositivity was associated with a greater percentage and number of senescent CD4+ T‐cells after exercise compared to their CMV seronegative counterparts (CMV+: 108,111,940 ± 30,105,496 cells vs. CMV‐: 86,502,865 ± 23,447,995 cells, *p* < 0.04).

**TABLE 3:** Wilcoxon matched pairs signed‐rank test from before (resting) and after (post) exercise on CD4+ and CD8+ T‐cell subset numbers

Cell subset (cell/ml)	*n*	Resting	Post	Z	*p*‐value
T Cells					
CD4+	21	816,763 ± 225,497	1,080,024 ± 355,575	−3.771	**<0.001**
CD8+	21	535,101 ± 203,487	779,485 ± 417,149	−3.702	**<0.001**
KLRG1‐CD57‐					
CD4+	15	72,659,125 ± 22,491,389	96,587,101 ± 28,068,784	−3.124	**0.002**
CD8+	15	29,767,819 ± 12,322,011	40,006,282 ± 23,693,745	−2.556	**0.011**
KLRG1+CD57+					
CD4+	15	4892 ± 5947	11,555 ± 20,978	−1.647	0.100
CD8+	15	41,098 ± 72,288	75,667 ± 127,611	−2.726	**0.006**
GLUT1+ T Cells					
CD4+	20	4180 ± 5316	3624 ± 3352	−0.261	0.247
CD8+	20	2753 ± 4170	3978 ± 4491	−1.157	0.794
GLUT4+ T Cells					
CD4+	13	4710 ± 2361	7197 ± 4149	−1.992	**0.046**
CD8+	14	3737 ± 2501	6185 ± 5804	−1.664	0.096
CD36+ T Cells					
CD4+	20	157,015 ± 177,718	190,275 ± 167,267	−0.896	0.370
CD8+	20	53,147 ± 52,869	55,755 ± 53,273	−0.075	0.940
HK1+ T Cells					
CD4+	13	43,054 ± 131,997	19,251 ± 56,979	−2.201	**0.028**
CD8+	15	62,459 ± 77,894	68,033 ± 91,001	−0.398	0.691
HK2+ T Cells					
CD4+	13	41,322 ± 128,528	45,999 ± 147,437	−0.175	0.861
CD8+	15	56,906 ± 89,599	59,879 ± 75,206	−0.398	0.691

Note

Values are mean ± SD; Bold indicates *p* < 0.05 are statistically significant.

### 3.3 Effect of exercise on T‐cell activation

**FIGURE 1:**
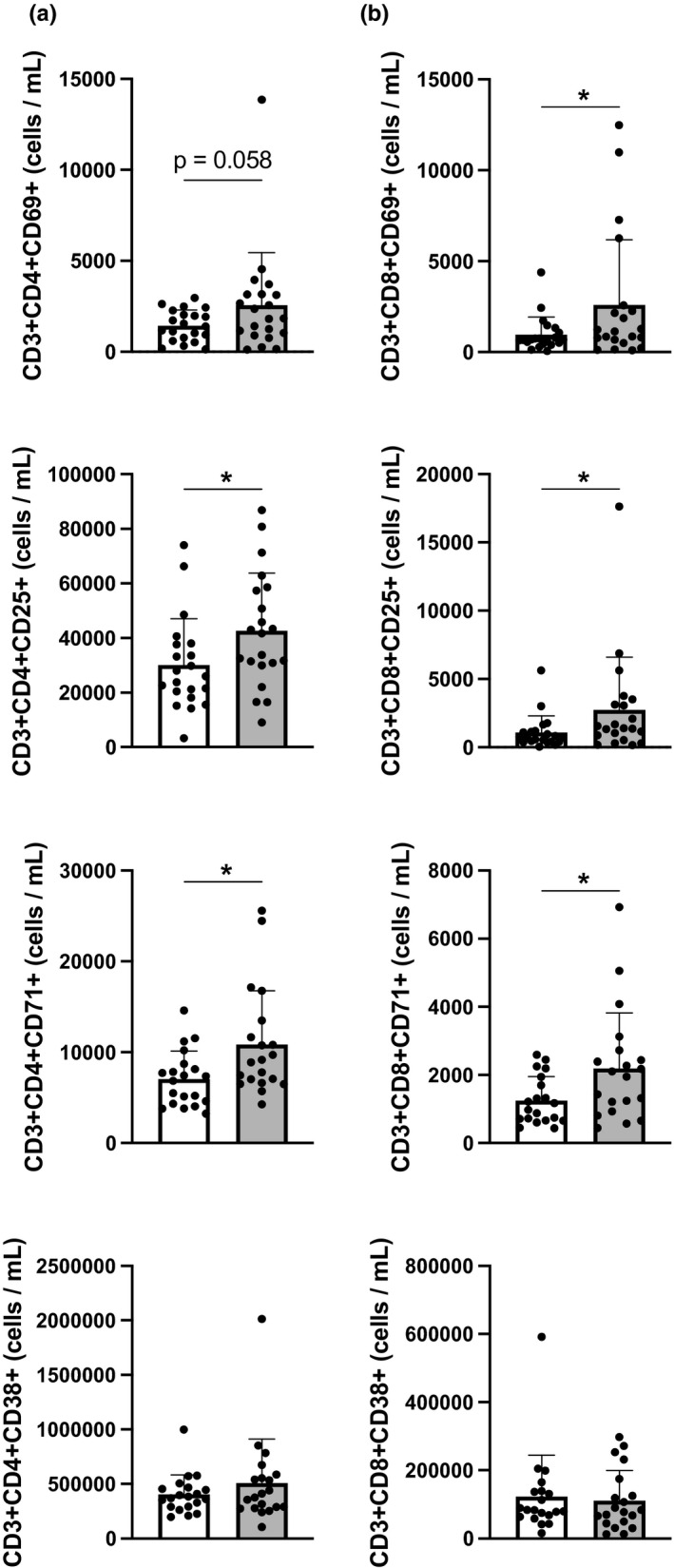
Resting and postexercise number of CD4+ (a) and CD8+ (b) T‐cells (*n* = 21). White bar indicated resting; gray bar indicated post‐exercise. Resting different from post‐exercise * *p* < 0.05 using Wilcoxon matched pairs signed‐rank test. Data are mean ± SD

Figure 1 presents the CD4+ and CD8+ expression of activation markers at rest and post‐exercise across all participants. The number of CD8+ T‐cells expressing very early (CD69; (Reddy et al., 2004)) activation markers were increased postexercise compared to the resting sample; while an increase in the number of T‐cells expressing early (CD25; (Reddy et al., 2004)) and late (CD71; (Motamedi et al., 2016)) activation markers was seen in both CD4+ and CD8+ T‐cells (*p* < 0.05). There was an increase in the percentages of CD4+ and CD8+ T‐cells expressing another late activation marker (CD38; (Motamedi et al., 2016)) after the exercise bout, but no difference was seen in the number of CD4+CD38+ or CD8+CD38+T‐cells (*p* > 0.05). Females had a higher percentage of CD8+CD38+ T‐cells at baseline compared to males; however, no sex differences were seen for cell numbers at baseline nor were any differences noted after exercise. The participant's sex had no effect on any of the other T‐cell activation markers studied here. CMV seropositive participants displayed a greater number of CD8+ T‐cells expressing very early, early, and late (CD71) activation markers after exercise compared to their CMV seronegative counterparts (*p* < 0.05).

### 3.4 Associations between T‐cell activation status and nutrient sensors in response to exercise

**FIGURE 2:**
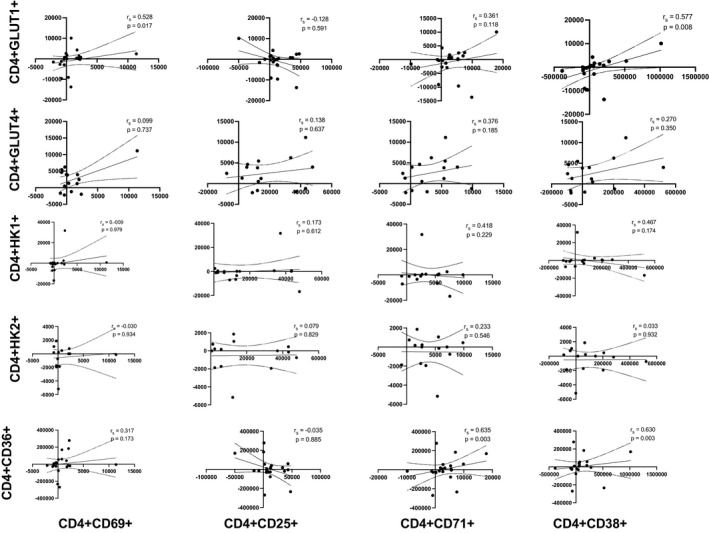
Scatterplot showing Spearman correlations of ingress (number of cells in the blood postexercise number of cells in the blood pre‐exercise) between activation markers (CD69, CD25, CD71, and CD38) and nutrient sensors (GLUT1, GLUT4, HK1, HK2, and CD36) in CD4+ T‐cells numbers

**FIGURE 3:**
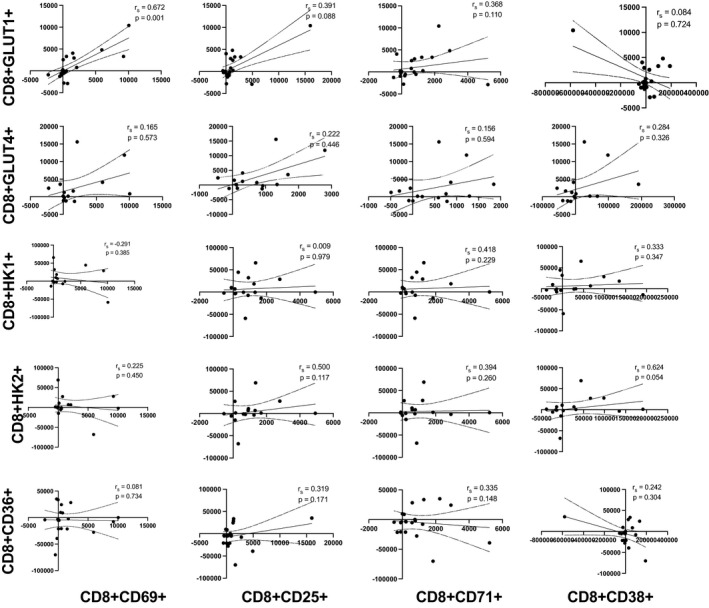
Scatterplot showing Spearman correlations of ingress (number of cells in the blood postexercise number of cells in the blood pre‐exercise) between activation markers (CD69, CD25, CD71, and CD38) and nutrient sensors (GLUT1, GLUT4, HK1, HK2, and CD36) in CD8+ T‐cells numbers

Cellular activation ultimately results in effector functions, requiring metabolic energy; therefore, we aimed to determine whether the relationship between the number of T‐cells that expressed activation markers and nutrient sensors for all participants. Activation markers (CD69, CD25, CD71, and CD38) were plotted against nutrient sensors (GLUT1, GLUT4, HK1, HK2, and CD36) in Figures 2 and 3. The number of CD4+ and CD8+ T‐cells expressing activation markers were sporadically correlated to the number of CD4+ and CD8+ T‐cells expressing GLUT1 pre‐ and post‐exercise (data not shown), and these relationships remained significant when correlations of the absolute number of cells mobilized into the blood (post‐exercise ‐ resting) were examined. The number of CD8+ T‐cells expressing very early (CD8+CD69+: *r*_s_ = 0.672, *p* = 0.001) and early (CD8+CD25+: *r*_s_ = 0.391, *p* = 0.088) activation markers mobilized with exercise were positively correlated with the number of CD8+GLUT1+T‐cells. A greater number of very early (CD4+CD69+: *r*_s_ = 0.528, *p* = 0.017) and late‐activated CD4+ T‐cells (CD4+CD38+: *r*_s_ = 0.577, *p* = 0.008) was positively correlated with the number of CD4+GLUT1+ T‐cells. The number of CD4+ T‐cells expressing late‐activated CD4+ T‐cells (CD4+CD38+: *r*_s_ = 0.556, *p* = 0.039) mobilized with exercise was positively correlated with the number of CD4+HK1+ T‐cells. Additionally, the number of CD4+ T‐cells expressing late (CD4+CD71+: *r*_s_ = 0.635, *p* = 0.003; CD4+CD38+: *r*_s_ = 0.630, *p* = 0.003) was positively correlated with the number of CD4+CD36+ T‐cells. No correlation was seen between CD8+ T‐cell activation markers and CD8+CD36+ (*p* > 0.05).

### 3.5 Mitochondrial ATP production rates in lymphocytes

**FIGURE 4:**
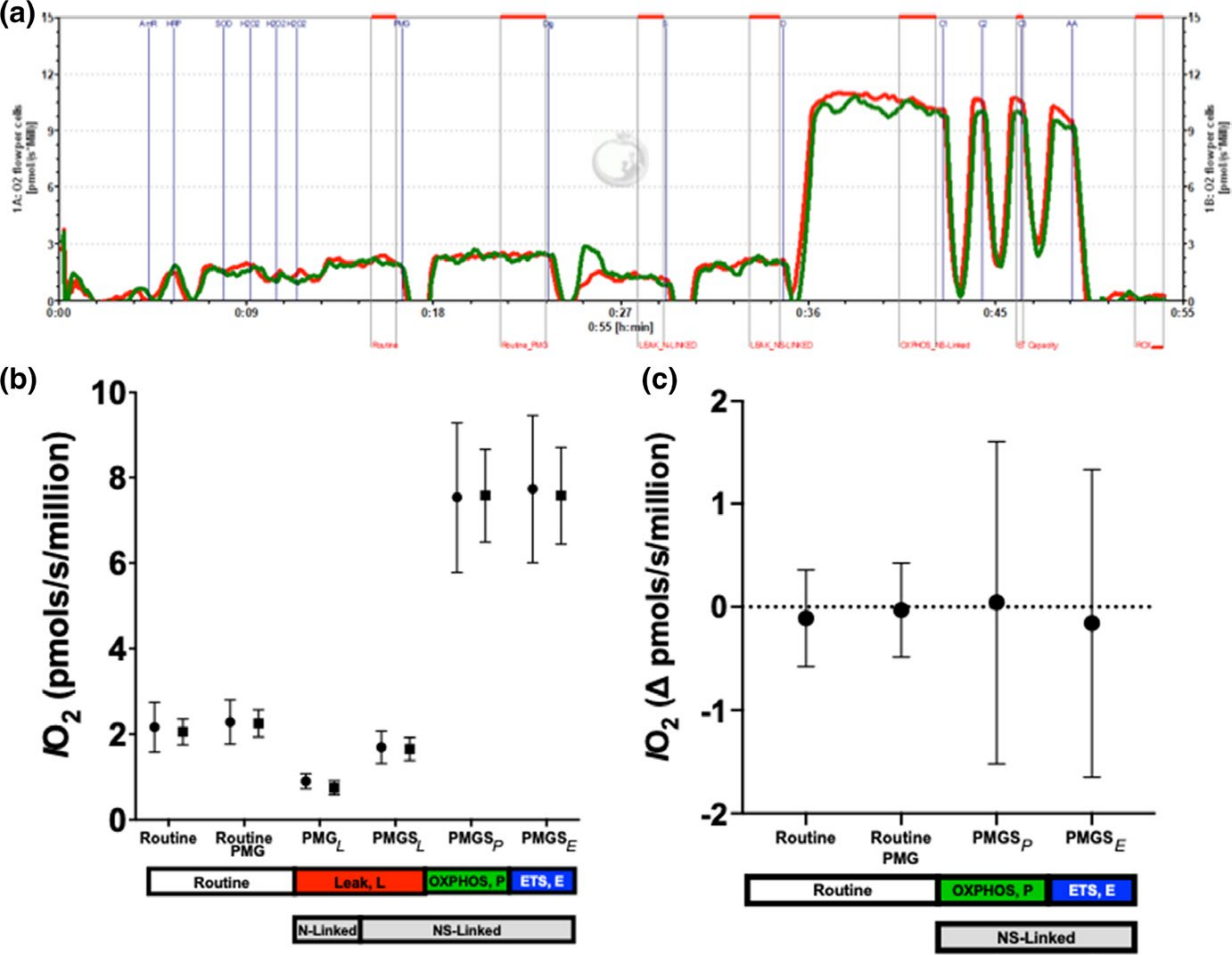
Panel (a) shows duplicate measurements (red line chamber A, green line chamber B) of oxygen flow (*IO_2_*, pmols/(s*million)) in Peripheral Blood Mononuclear Cells (PBMCs) acquired at 37°C in mitochondrial respiration media (MiR05‐kit, pH = 7.1) from a representative experiment. Panel (b) shows the mean (95% confidence interval) resting (•) and post‐exercise (■) *IO_2_* under routine, LEAK, OXPHOS, and ETS capacity respiratory states for the total sample. Routine respiration was recorded in intact PBMCs before and after adding pyruvate (P, 5 mM), malate (M, 2 mM), and glutamate (G, 10 mM). NADH‐linked (N‐linked) LEAK respiration (LEAK_N_) was recorded after adding digitonin (7.5–10 μg/10^6^ cells) in the absence of ADP. NADH plus succinate‐linked (NS‐linked) LEAK respiration (LEAK_NS_) was recorded after adding succinate (S, 10mM) in the absence of ADP. NS‐linked oxidative (OXPHOS_NS_) capacity was recorded after adding a saturating concentration of ADP (D, 5 mM). NS‐linked maximal uncoupled respiration rate associated with ETS capacity was recorded after serial titrations of carbonyl cyanide m‐chlorophenyl hydrazine (CCCP, ~1 μM). Finally, we added 2.5 µM antimycin A to measure residual oxygen consumption, which was subtracted from the routine, LEAK, OXPHOS, and ET capacity rates to isolate the mitochondrial oxygen consumption rates during these respiratory states. Panel (c) shows the mean (95% confidence interval) respiratory control ratios (RCR, OXPHOS_NS_/LEAK_NS_) measured in the resting and postexercise samples for the total sample

**FIGURE 5:**
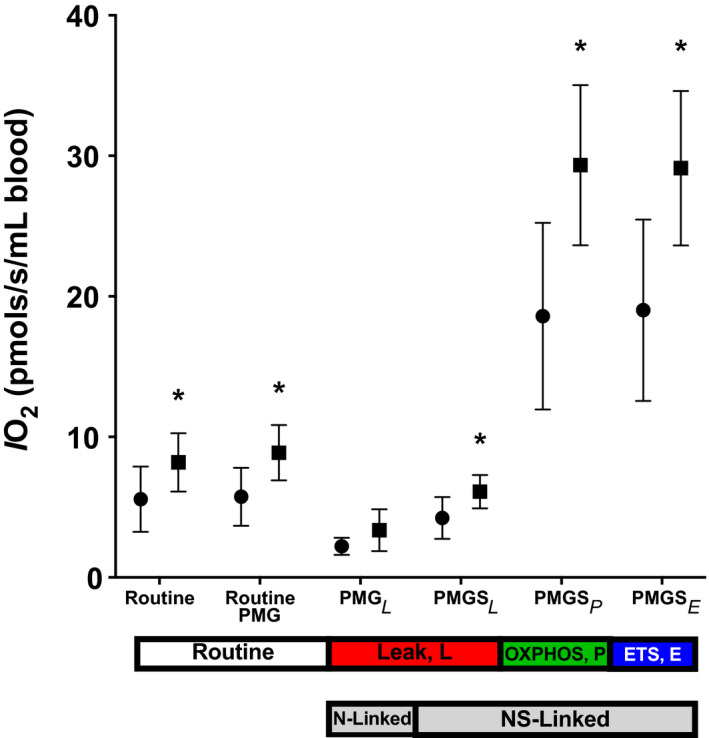
Mean (95% confidence interval) resting (•) and post‐exercise (■) oxygen flux normalized per mL of blood (IO2‐tissue, tissue level normalization) under routine, LEAK, OXPHOS, and ETS capacity respiratory states for the total sample. * Differences between pre‐ and post‐exercise, *p* < 0.05

One of the study's goals was to assess whether changes in PMBC mitochondrial respiratory function occur in response to an acute bout of exercise. Figure 4 shows the mitochondrial respiration rate per million PBMC (*IO_2_*) at rest and following the acute bout of exercise. An example of the oxygraph experiment protocol is displayed in Panel A. The mitochondrial *IO_2_* did not change in response to the acute exercise (Figure 4b). Additionally, the mitochondrial respiratory control ratio (RCR, OXPHOS_NS_/LEAK_NS_) also did not change in response to the acute bout of exercise (Figure 4c). However, since exercise acutely increases the concentration of PBMCs in the blood (tissue), we also analyzed the mitochondrial respiration rates per mL of blood (tissue), accounting for the exercise‐induced increase in PBMC concentration in an mL of blood. When mitochondrial respiration rates were analyzed in this tissue level fashion, we noted increases in respiration rates post‐exercise, including both routine and mitochondrial oxidative capacity (Figure 5). Sex and CMV serostatus also did not affect any exercise‐induced increases in respiration rates when analyzed at either the cellular or tissue level.

## 4 DISCUSSION

The primary findings of the present study are (i) that activated T‐cells primed for glucose uptake are preferentially mobilized with exercise, (ii) PBMC mitochondrial respiratory function *at the cellular level* did not increase following an acute bout of moderate‐to‐vigorous exercise when normalized per million cells (i.e., no change in a specific activity of PBMC mitochondrial respiratory function for individual cells), and (iii) PBMC mitochondrial respiratory function increased *at the tissue level* following an acute bout of moderate‐to‐vigorous intensity exercise when normalized per ml blood (i.e., increase in activity of PBMC mitochondrial respiratory function for all cells in circulation).

Cellular activation in response to mitogen‐induced stimulation has been well characterized in T‐cells (Motamedi et al., 2016; Reddy et al., 2004). However, little is known about the activation response to exercise. Studies have documented an increase in very early activated (CD3+CD69+) and early (CD3+CD25+) CD4+ and CD8+ T‐cells following exercise (Curran et al., 2019; Morabito et al., 2016). The present results confirm and extend these findings by showing an increase in late‐activated CD4+CD71+ and CD8+CD71+ T‐cells (Figure 1).

Activated immune cells largely depend on a glycolytic metabolism to fuel rapid ATP production for proliferation and cytokine production (Pearce & Pearce, 2013). For this reason, activated T‐cells increase the expression of glucose transporters to meet glucose requirements (MacIver et al., 2013). Acute exercise, a cellular stress, can activate T‐cells (Curran et al., 2019); however, the impact that acute exercise, such as 30‐min of moderate‐to‐vigorous intensity exercise, has on T‐cell nutrient‐sensing mechanisms is largely unknown. To investigate this relationship, we examined the effects of exercise on CD4+ and CD8+ subsets expression of nutrient sensors using flow cytometry (Table 3). While some statistically significant changes were noted for an exercise‐induced increase in the number of cells expressing glucose transporter 4 (GLUT4) and an exercise‐induced decrease in the number of cells expressing the glycolytic enzyme, hexokinase 1 (HK1), in CD4+ T‐cells, we consider these results as explorative due to the low sample size. The relationship between glucose transporters and T‐cells has been previously explored in vitro‐stimulated CD4+ T‐cells, finding activated T‐cells do not express GLUT4 and instead depend on GLUT1 upregulation (Macintyre et al., 2014). The difference in findings between our study and the aforemention research may result from differences in stimulation (in vitro vs. in vivo) or outcome measure (mRNA expression vs. surface protein expression). Our findings also support previous research suggesting HK1 mRNA levels are reduced in CD4+ T‐cells cultured with postexercise serum (Palmowski et al., 2021). Since reductions in HK1 would likely be associated with reduced glucose‐6‐phosphate formation and concomitantly lower glucose uptake, we hypothesize that this could shift cellular metabolism toward fatty acid uptake. Although we did a positive correlation between exercise‐induced CD4+ T‐cell activation and increased expression of the fatty acid transporter CD36, the overall number of CD4+ T‐cell expressing CD36 post‐exercise was not statistically different from the pre‐exercise values. Instead, our data showed that absolute numbers of CD4+ and CD8+ T‐cells expressing activation markers were often significantly correlated to the absolute number of CD4+ and CD8+ T‐cells expressing glucose transporters. Although exploratory, these findings could highlight a compensatory mechanism where activated CD4+ T‐cells that were preferentially mobilized with exercise would upregulate GLUT‐4 to counteract the reduced expression of HK1. Further investigation is required to determine whether the increased expression of these nutrient transporters tanslates into increased glucose uptake and/or glucose utilization in PBMC following moderate‐to‐vigorous exercise.

There is emerging interest in the acute and chronic effects of exercise on immune cell mitochondrial bioenergetics (Busquets‐Cortes et al., 2017; Rosa et al., 2021). The present results suggest that a 30‐minute moderate‐to‐vigorous intensity exercise session did not significantly affect PBMC mitochondrial respiratory function when normalized per million cells in our cohort of college‐aged adults. In contrast, a recent study reported that a 1‐hour bout of low‐intensity exercise increased routine respiration rates as well as the respiration rates in the LEAK and OXPHOS states when oxidizing palmitoyl carnitine in middle‐aged adults (Liepinsh et al., 2020). In the present study the routine respiration rate under the pre‐exercise conditon was slightly lower than those presented in the aformentioned study (~ 2 vs. ~4 pmols/(s*million)). The longer exercise duration (1‐h vs. 30 min) could have contributed to the stimulation of mitochondrial respiration rates, particularly those associated with fatty acid oxidation in the PBMCs observed in their middle‐aged adults. As stated previously, an in vitro study also showed no significant changes in oxygen consumption rates after culturing CD4+ T‐cells with postexercise serum (Palmowski et al., 2021), which also suggests that the postexercise milieu does not necessarily stimulate PBMC respiratory function. Therefore, while there is a preferential mobilization of activated immune cells in the blood in response to exercise, this may not translate to changes in nutrient‐sensor expression or mitochondrial changes in PBMCS of young, healthy adults.

Often in mitochondrial experiments, acute increases in mitochondrial respiration are assumed to be due to an acute activation of the mitochondria that are already present in the tissue of interest and in the absence of acute changes in mitochondrial content within the tissue of interest (e.g., skeletal muscle). However, intensive exercise causes a significant increase in circulating lymphocytes (Spielmann et al., 2014) and monocytes (Rooney et al., 2018; Simpson et al., 2009) and, consequently, a procedure normalizing respiration rates per million PBMCs ignores this biologically relevant increase in circulating PBMCs (i.e., exercise‐induced leukocytosis). Although, the present results showed no significant differences in the respiration rates on a per cell basis in response to exercise, we found increased respiration rates in response to exercise after adjusting to the increased concentration of PBMCs in the blood (tissue). We hypothesized this increase in respiration at the *tissue level* would result in increased T‐cell and/or monocyte function and increase systemic immunosurveillance; however, this idea requires further extensive investigation which should include measurements of cytokine secretion and associated effector functions. In addition, although it can be hypothesized that changes in PBCM OXPHOS in response to acute exercise could ultimatley lead to improvements in systemic metabolism if performed regularly (i.e., exercise training), this remains to be tested. To our knowledge this is the first study to document an exercise‐induced increase in mitochondrial respiration at the *tissue level*. Our data are also consistent with increased expression of glucose transporters in activated T‐cells (Maratou et al., 2007), therefore, increased glucose uptake may explain our observed increase in respiration for PBMCs while in circulation. We believe these results highlight an exciting potential explanation for how frequent exercise impacts systemic metabolism. Future studies are needed to determine glucose uptake rates in PBMCs after exercise.

In a recent review, Duggal et al. (2019) theorized that exercise increases immunosurveillance and, therefore, improves control of latent viral infections (Duggal et al., 2019). CMV infection has been shown to exacerbate features of immune aging (Fulop et al., 2013). Previous research suggests lifelong exercise mitigates the accumulation of highly differentiated T‐cells (Spielmann et al., 2011), yet the mechanism of this change remains unknown. Therefore, we decided to investigate the effect of CMV serostatus in this acute exercise study. Greater exercise‐induced mobilization of senescent CD4+ T‐cell populations in CMV+ participants reflect previous findings, but the similarities in senescent CD8+ T‐cells between CMV+ and CMV− participants was surprising, especially given previous research showing resting and exercise‐induced differences in this age group (Theall et al., 2020; Turner et al., 2014). We also did not find any effect of CMV on mitochondrial respiration at rest or after exericse. The study's population of young, healthy adults could explain the lack of mitochondrial deterioration within the CMV+ participants. Mitochondrial respiration is known to decrease with age (McGuire, 2019; Sun et al., 2016), particularly in those with cardiometabolic diseases (Chistiakov et al., 2018; Dai et al., 2012) and frailty (Chistiakov et al., 2014; Sonjak et al., 2019), therefore, a difference in mitochondrial function may not have occurred in this population. Additionally, we do not know when the participants initially acquired CMV, so we cannot determine whether the amount of time the participant had been infected or whether or not these participants were actively undergoing CMV reactivation affected our results. Our results suggest acute exercise increases the number of activated, respiring T‐cells in circulation, potentially increasing immunosurveillance and reducing the need for large quantities of highly differentiated T‐cells. Given lifelong exercise is an accumulation of acute exercise bouts, exercise‐induced increases in activated T‐cells may explain why features of immunosenescence are not consistently seen in CMV+ physically active individuals (Bartlett & Duggal, 2020). However, the effects of chronic exercise on T‐cell activation, nutrient sensing, and metabolic function in CMV seropositive participants require further investigation.

A particular strength of this study is the use of flow cytometry and high‐resolution respirometry to assess immediate and direct changes in T‐cell phenotypes and PBMC mitochondrial respiratory function before and after an acute bout of exercise, which extends previous research using cell culture models with oxygen sensors (Palmowski et al., 2021). Nevertheless, there are some caveats and limitations to this study worth discussing. First, our population was made up of young, healthy participants; therefore, we cannot extrapolate our results to an older, diseased populations. While our study included female participants, we did not control the menstrual cycle or hormonal contraceptive use which could have an impact on PBMC mitochondrial respiraotry function. In addition, our study was also not powered to detect sex differences in PBMC phenotypes or mitochondrial respiratory function. That said, recent data suggest that there may be some sexual dimorphism in PBMC mitochondrial respiratory function (Silaidos et al., 2018). We also note that the OXPHOS and ET Capacity rates in the present study (~8 pmols/(s*million)) was higher than some previous reports (~3–4 pmols/(s*million)) (Rose et al., 2019), but lower than others (~14–18 pmols/(s*million)) (Hedges et al., 2019; Hsiao & Hoppel, 2018). A potential explantion for lower values for OXPHOS and ET Capacity could be related changes in endogenous substrates, specifically glutmate, which has been reported reduce respiratory rates in PBMCs in some (Rose et al., 2019) but not all reports (Hsiao & Hoppel, 2018). Future studies may investigate the effect of these factors on PBMC respiratory function, particularly in purified T‐cells and monocytes. It could also be noted that while we chose to measure OXPHOS using high‐resolution respirometry, other measures of metabolic functions such as extracellular flux analysis and glucose uptake assay could have been better suited to estimate glycolytic function in mixed PBMCs post‐exercise. Although we were unable to include measures of H_2_O_2_ production in our immune cells studies, future studies investigating the acute effect of exercise on the both mitochondrial and non‐mitochondrial reactive oxygen species (e.g., H_2_O_2_) production are warranted. Moreover, the addition of inhibitors of the antioxidant defense systems (e.g., Auronafin and Dinitrochlorbenzene) may be needed to accurately assess the production of reactive oxygen species within the mitochondria of immmune cells, similar to what has been reported in mitochondria from muscle (Fisher‐Wellman et al., 2018; Munro et al., 2016). We also did not account for shifts in monocyte populations which may have impacted our results. In addition, future studies should also examine the impact of acute exercise on the ability to switch between mitochondrial fuel sources (e.g., fatty acids vs. carbohydrates) are needed, along with studies assessing changes in nutrient uptake. Finally, given the complexity of the PBMC pool, future studies should also focus on assessing changes in nutrient sensing and mitochondrial respiratory function in isolated T‐cell subsets (e.g., CD4+ and CD8+ T‐cells).

Overall, our results suggest that exercise leads to an increase in the circulating concentration of PBMCs (e.g., exercise‐induced luekocytosis), particularly with respect to activated T‐cells. Transient increases in cells that are primed for glucose uptake (e.g., greater localization of GLUT4 on the cell surface) may improve their ability to utilize glucose, which in turn would alter their effector functions in response to acute exercise. Although exercise did not appear to stimulate PBMC respiratory function at the *cellular level* as we hypothesized, exercise did increase the mitochondrial respiratory function (e.g., routine and OXPHOS_NS_) at the *tissue level*. Future studies are needed to assess how these changes in nutrient sensing and increases in *tissue level* mitohcondrial respiratory function can affect immune function in response to acute exercise, which may help to explain how chronic exercise is known to positively influence immune function with aging.

## CONFLICT OF INTEREST

The authors declare no conflict of interest.

## AUTHOR CONTRIBUTIONS

Conceptualization and methodology, G.S., B.A.I., and N.M.J.; investigation, B.T., E.C., J.S., and J.G.; writing—original draft preparation, B.T.; writing—review and editing, B.T., E.C., J.S., J.G., N.M.J., B.A.I., and G.S. All authors have read and agreed to the published version of the manuscript.

## Supporting information



Table S1
