# Supplementary material for: Impact of acute exercise on peripheral blood mononuclear cells nutrient sensing and mitochondrial oxidative capacity in healthy young adults
Source: Physiol Rep. 2021 Dec 10;9(23):e15147. doi: 10.14814/phy2.15147 (PMC8661513; doi:10.14814/phy2.15147)
Supplement: Supplementary file 1 — Table S1 [file PHY2-9-e15147-s001.docx]

**Table S1. Phenotypic identification of T-cell subsets.**

|  |  |
| --- | --- |
| **Cell Type** | **Phenotypic identification** |
| T-cells | CD3+CD4+CD8- or CD3+CD4-CD8+ |
| Level of Differentiation |  |
| Early Differentiated | KLRG1-/CD57- |
| Mid- Differentiated | KLRG1+/CD57- |
| Highly Differentiated | KLRG1+/CD57+ |
| Activated T-cells |  |
| Very early | CD69+ |
| Early | CD25+ |
| Late | CD71+ or CD38+ |
| Nutrient Sensors |  |
| Glucose transporters | GLUT1+ and GLUT4+ |
| Fatty acid translocase | CD36+ |
| Glycolytic enzymes | HK1+ and HK2+ |
